# What happens next? Repeated administration of a social and structural determinants of health questionnaire in an Alzheimer’s Disease Research Center

**DOI:** 10.1002/alz.092745

**Published:** 2025-01-09

**Authors:** Shana D. Stites, Valerie Humphreys, Carolyn Kuz, Rosalie Schumann, Kimberly Halberstadter, Dawn Mechanic‐Hamilton

**Affiliations:** ^1^ Department of Psychiatry, Perelman School of Medicine, University of Pennsylvania, Philadelphia, PA USA; ^2^ University of Pennsylvania, Philadelphia, PA USA

## Abstract

**Background:**

Social and structural determinants of health (SSDoH) must be measured longitudinally in order to better understand the effects of lived experiences on the trajectories of Alzheimer’s disease and related dementias (ADRD) outcomes. We repeated administration of an SSDOH survey to examine response rates and response consistency in a sample of cognitively typical older adults.

**Method:**

One online survey and one follow‐up survey were administered to cognitively unimpaired participants (n = 135) in the UPenn Alzheimer’s Disease Research Center clinical cohort ∼1.5 years apart. The ∼225 items covered the following categories: (1) Education, (2) Economic Status and Strain, (3) Occupation, (4) Stressors and Subjective Stress, (6) Subjective Social Support and Status, and (7) Sociodemographic data, which included age, race, language, disability, neighborhood, sex, sexual orientation, and gender identity questions. We summarize pilot data on item and instrument completion rates and changes in participant responses.

**Result:**

81 of 135 participants completed the follow‐up survey, which is a response rate of 60%. Most participants completed most items. The two measures with the lowest completion rates were: Calgary Charter on Health Literacy Scale (74/81) and the Perceived Stress Scale (69/81). Some participants who did not complete a given measure during the first administration did complete that measure during the second. In addition, responses on most measures, single items or multi‐item scales, varied between the administrations. Some responses appeared to change because of ambiguity in the item’s phrasing, administration process, or reflected changes over time in the constructs being assessed.

**Conclusion:**

Overall, it was feasible to repeatedly administer a survey of SSDoH items to a group of cognitively unimpaired older adults. The response rate was reasonable but lower than expected in a group of volunteer research participants indicating that multiple modes of administration may be useful for increasing engagement. Differences in the data produced from repeated administration of measures was useful in identifying ambiguity in items and methods that aid in improving research rigor and reproducibility. The data are also useful in understanding the temporal stability or instability of SSDOH experiences among these research participants.

